# Parachute Mitral Valve in an Adult

**DOI:** 10.14797/mdcvj.1193

**Published:** 2023-01-20

**Authors:** Priscilla Wessly, William A. Zoghbi

**Affiliations:** 1 Houston Methodist DeBakey Heart & Vascular Center, Houston, Texas, US

**Keywords:** parachute mitral valve, mitral stenosis, congenital heart disease

## Abstract

Two-dimensional transthoracic echocardiography images for a 49-year-old female with a history of ventricular septal defect status post repair, type 2 diabetes mellitus, and hyperlipidemia whose evaluation of her lower extremity edema showed parachute mitral valve.

Parachute mitral valve is a rare congenital anomaly characterized by unifocal attachment of the chordae tendinea of anterior and posterior mitral valve leaflets to a single papillary muscle. They are thought to develop due to disturbed lamination of the anterior and posterior part of the trabecular ridge, which normally forms the anterolateral and posterolateral papillary muscles respectively between the 5th and 19th week of gestation.^1^ It rarely occurs as an isolated lesion and is usually associated with other congenital cardiac anomalies. The outcomes are dependent on the spectrum of associated lesions.^2^ The pathognomonic pear-shaped view is seen in the apical long views on transthoracic echo, with the atrium forming the base of the pear and mitral valve leaflets forming the apex. The chordae tendinea are often short and thick. This, coupled with the convergent papillary insertion, leads to decreased mobility of the valve leaflets, thus causing stenosis. In adults, parachute mitral valves are rare and can be asymptomatic or associated with mild stenosis.^3^

Figure 1 shows transthoracic echocardiography 2-dimentional images of a 49-year-old female with a history of ventricular septal defect status post repair, type 2 diabetes mellitus, and hyperlipidemia. She underwent transthoracic echocardiogram for evaluation of her lower extremity edema. Transthoracic echocardiography shows parachute mitral valve.

**Figure 1:**
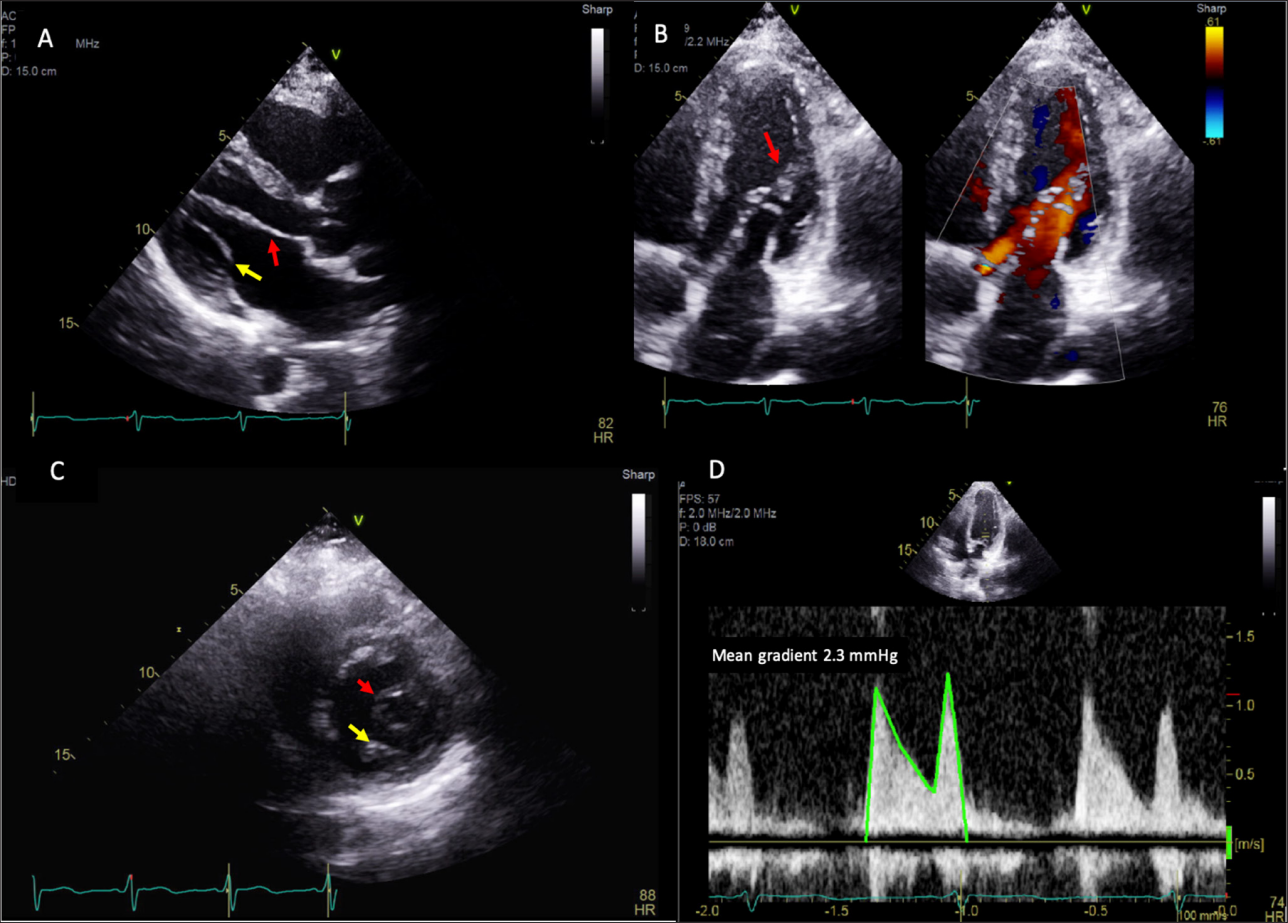
**(A)** Parasternal long view on transthoracic echo showing thickened anterior (red arrow) and posterior mitral valve (yellow arrow) leaflets. **(B)** Apical long-axis view showing pear-shaped eccentric opening of the mitral valve with short chordae tendinea attached to the anterolateral papillary muscle (red arrow) and transmitral flow during diastole. **(C)** Parasternal short-axis view showing an eccentric mitral valve orifice (red arrow) predominately oriented towards the anterolateral papillary muscle and a remnant of the posteromedial papillary muscle (yellow arrow). **(D)** Spectral Doppler showing mean gradient of 2.3 mm Hg at a heart rate of 74 beats/min across the mitral valve in diastole.
